# Veterinary Drug Residues in Food Products of Animal Origin and Their Public Health Consequences: A Review

**DOI:** 10.1002/vms3.70049

**Published:** 2024-09-27

**Authors:** Yeshambel Muluye Mesfin, Birhan Agmas Mitiku, Habtamu Tamrat Admasu

**Affiliations:** ^1^ Amhara Agricultural Research Institute Bahir Dar Ethiopia; ^2^ Department of Veterinary Science College of Agriculture and Environmental Sciences Bahir Dar University Bahir Dar Ethiopia

**Keywords:** antimicrobial resistance, drug residues, food safety, public health

## Abstract

Veterinary medications used for disease treatment and prevention may remain in animal‐origin foods, such as milk, eggs, honey and meat, which could pose a risk to the public's health. These drugs come from different groups of drugs, mostly with antibiotic, anti‐parasitic or anti‐inflammatory actions, in a range of food matrices including milk, meat or egg. This review is intended to provide the reader with a general insight about the current status of veterinary drug residues in food products of animal origin, detection methods and their public health consequences. The discovery of antimicrobials has led to the development of antibiotics for treating and preventing cattle illnesses and encouraging growth. However, the rise of drug resistance has led to increased antibiotic consumption and resistance among microbes in the animal habitat. This resistance can be passed to humans directly or indirectly through food consumption and direct or indirect interaction. Improper and illegal use, inadequate withdrawal periods and environmental contamination from veterinary drugs are reported to be the major causes for the formation of residue in food products of animal origin. The use of veterinary products above or below the advised level may also result in short‐ or long‐term public health issues, such as the creation of resistant strains of micro‐organisms, toxicity, allergy, mutagenesis, teratogenicity and carcinogenetic effects. To ensure consumer safety, veterinary drug residues in food must be under control.

## Graphical abstract

Improper and illegal use, inadequate withdrawal periods and use of banned drugs in veterinary medications may remain in animal‐origin foods. Numerous studies confirmed that there are abundant residues of veterinary drugs in animal‐origin foods that could cause public health risks. To prevent veterinary drug residues in food, evidence‐based One Health approach interventions are per amount important.

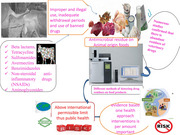

## 1 Introduction

People's health is closely related to environmental safety, particularly the type and calibre of food that they consume. To protect animal health, medicines and other substances are used in the production of livestock and poultry (Bahmani, Shahbazi, and Nikousefat 2020; Dowling 2013). Veterinary medications used for disease treatment and prevention may remain in animal‐derived foods such as milk, eggs, honey, meat or organs after slaughter coming from treated animals, which could pose a risk to the public's health (Novaes et al. 2017; Ortelli, Spörri, and Edder 2018). Many factors, including pharmacological qualities and pharmacokinetics, and physical, chemical or biological processes involving animals and their products, can affect the presence of residues in animal products. The primary causes of drug leftovers are improper drug use and disregard for the withdrawal period (Beyene et al. 2015). Currently, there are over 200 veterinary medication residues from different families, mostly with antibiotic, anti‐parasitic or anti‐inflammatory actions, in a range of food matrices, including milk, meat or egg (Delatour et al. 2018).

One of the most important discoveries in medical history is the discovery of antimicrobials. Their application in veterinary care and animal husbandry has produced farm animals that are healthier and more productive, ensuring the welfare and health of both animals and people. Antibiotics are frequently used to treat and prevent cattle illnesses as well as to encourage growth (Economou and Gousia 2015; Owusu‐Doubreh, Appaw, and Abe‐Inge 2023).

The advent of problems with drug resistance, however, led governments to pass a number of rules governing the use of antibiotics in livestock. Antibiotic usage and abuse in farm animal environments, whether as growth promoters or as general methods of illness prevention and treatment, has increased antibiotic consumption and resistance among microbes in the animal habitat (Ghimpețeanu et al. 2022; Menkem et al. 2019). Through food consumption and direct or indirect interaction, this reservoir of resistance can be passed to humans directly or indirectly (Economou and Gousia 2015; Xie et al. 2018). The use of veterinary products above or below the advised level might result in short‐ or long‐term public health issues, such as the creation of resistant bacterial or parasitic strains, toxicity, allergy, mutagenesis, teratogenicity and carcinogenetic effects. Various authorities throughout the world have implemented measures to reduce antimicrobial use (AMU) in livestock production due to the growing concern regarding the evolution of bacteria resistant to antibiotics and their potential for transmission to people through animal agriculture (Atta et al. 2022; Lhermie, Gröhn, and Raboisson 2017). Following control of the problem of drug‐resistant bacteria, public attention has shifted to the recurring incidence of human health and safety issues caused by residual veterinary drugs in livestock products (Chen, Ying, and Deng 2019; Lee et al. 2018).

Without following the withdrawal period, the indiscriminate and illogical use of antibiotics in animals may leave unanticipated residues in food sources (meat, eggs and milk), which could pose major health risks to humans (Chowdhury et al. 2015; Mingle et al. 2021). As veterinary medications are used to treat animals for both preventive and therapeutic purposes, antibiotic residues may be identified in food that comes from animals. To ensure consumer safety, veterinary drug residues in food must be under control (Gaudin 2017).

Contrary to industrialized nations, developing nations have few, if any, programmes or procedures in place to keep an eye on the use of antibiotics by humans, animals used for food and other food‐producing items. The use of antibiotics in food animals has a considerable impact on antibiotic resistance, a serious health and socioeconomic issue that affects the entire world. Antibiotic resistance must be addressed equally in the developed and developing worlds if antibiotic resistance is to be successfully combated on a global scale (Founou, Founou, and Essack 2016; Ghimpețeanu et al. 2022).

In Ethiopia, there is practically no government oversight or supervision over the prudent use of veterinary medications. There is no reliable method for measuring the national antimicrobial level in animal food products, despite the widespread use of antimicrobials in food animals, especially antimicrobials, which pose a serious threat to food safety and public health (Beyene et al. 2015; Tesfaye 2019). Veterinary Drug and Feed Administration and Control Authority, which is accountable to the Ministry of Agriculture, is responsible for regulating the use and licensing of veterinary drugs; however, there are no standards in place (Aytenfsu and Mamo 2016). Therefore, the objective of this review is an overall insight about major veterinary drug residues in food products of animal origin and their public health consequences.

## 2 Method of Review

In this review, all information related to the topic of interest was obtained from a literature search of electronic databases such as Science Direct, Google Scholar, PubMed and BASE. The published studies on antibiotic residues in foods of animal origin and its public health implications were included. The search engines were accessed from March 2023 to June 2023 to search for original scientific works published in English from year 1999 to 2023. Most of the articles used for this review were published starting from year 2015. The following terms were used to search scientific works: (antimicrobial* antibiotic* veterinary drugs* drug residues* public health impact of drug residues* meat* poultry* eggs* beef* milk* honey* animal products* detection methods*). All retrieved records were saved, and publications not relevant to this review or written in languages other than English were excluded. Publications containing antimicrobial residues data in the abstract were selected for a first investigation. All retrieved scientific works that dealt with residues of veterinary drug residues and their consequences were included. Generally, around 107 published literatures related to this topic were used.

## 3 Major Veterinary Drugs for Residue Formation

Antimicrobial drugs are used therapeutically to protect both the health and welfare of people and animals. Antibiotic medications work by preventing micro‐organisms like bacteria, fungi, parasite or protozoa from reproducing or growing. There are over 250 different chemical compounds that have been authorized for use in human and veterinary medicine for treating, preventing and controlling illness as well as fostering growth (Kümmerer and Henninger 2003; Menkem et al. 2019).

The most often prescribed medications in veterinary medicine include antibiotics (β‐lactams, tetracyclines, fluoroquinolones, sulphonamides and macrolides), anti‐parasitic medications (avermectins and benzimidazole) and NSAIDs (non‐steroidal anti‐inflammatory drugs). Several dietary matrices of animal products, such as milk, meat and eggs, contain the veterinary drugs and are subject to regulation (Delatour et al. 2018; Rizzetti et al. 2017; Soares et al. 2022).

### 3.1 β‐Lactams

A class of medications known as β‐lactams has a hetero‐atomic β‐lactam ring nucleus with three carbon atoms and one nitrogen atom in the ring structure. When they act, they damage the bacterial cell walls (Menkem et al. 2019). The most popular class of antimicrobial medications for many years has been this one, which comprises penicillins, cephalosporins and carbapenems. Their widespread usage as growth promoters, chemotherapeutic and/or prophylactic medicines in veterinary care procedures results in various residues in foodstuffs that pose a severe health risk (Girmatsion et al. 2021; Lara et al. 2012; Msagati and Nindi 2007; Popelka et al. 2005; Zhang et al. 2020).

β‐Lactam residues may enter the food chain as a result of either illicit β‐lactam use or companies failing to follow current animal‐treatment guidelines (withdrawal times) before animal products are accessible for consumption (Kantiani et al. 2009). The synthesis of particular enzymes by bacteria, such as β‐lactamase, which breaks down the antibiotics’ four‐atom β‐lactam ring, is one of their ways for resisting antibiotics. The antibiotics’ antibacterial effects are fully lost when this ring is opened by these enzymes during hydrolysis (Tekiner and Özpınar 2016).

### 3.2 Tetracycline

Tetracycline, a class of antibiotics that includes oxytetracycline, tetracycline, chlortetracycline and doxycycline, is frequently used in the livestock sector due to its effectiveness against both gram‐positive and gram‐negative bacteria. Numerous infections are treated with tetracycline medication, and it also helps animals thrive (Girmatsion et al. 2021; Soares et al. 2022). This approach could lead to trace levels of these medications in animal‐derived goods, including milk, meat and eggs, posing significant dangers to human health. Tetracycline residues in food can cause antibiotic‐resistant pathogenic micro‐organisms to spread across the food chain (Gaurav et al. 2014; Pérez‐Rodríguez et al. 2018).

### 3.3 Quinolones and Fluoroquinolones

Fluoroquinolones and quinolones are broad‐spectrum drugs that work by inhibiting DNA gyrase, a crucial bacterial enzyme. They are frequently used to treat and prevent disease in both people and animals (Bucknall et al. 2003; Navrátilová et al. 2011). This class of antimicrobials includes chemically varied substances that have a 4‐quinolone ring structure. The antibacterial efficacy and pharmacokinetic features of the class have been improved through a variety of chemical changes involving the substitution of various functional groups around the quinolone ring (Ashwin et al. 2009).

Growing worry has been expressed about the significant risk to human health connected with extensive animal farming and animal usage of antibiotics due to the rise in microbial resistance to these medications (Gouvêa et al. 2015; Martins et al. 2015; Pena et al. 2010).

### 3.4 Sulphonamides

Sulphonamides are a class of synthetic antimicrobials that are effective against the majority of gram‐positive and gram‐negative bacteria as well as protozoa. Animals and humans both employ sulphonamides for medicinal and preventative purposes (Ramatla et al. 2017). Sulphadiazine showed approximately 10% loss during milk boiling. Sulphonamide residues are stable in animal tissues when frozen (Rana et al. 2019).

### 3.5 Macrolides

Macrolide antibiotics are a group of antibacterial substances that are effective against gram‐positive and some gram‐negative bacteria. They are frequently used to treat respiratory illnesses and enteric infections in cattle, sheep, pigs and poultry (Berrada et al. 2010; Tao et al. 2012). These antibiotics are important medications for the clinical treatment of bacterial infections and are added to feed to promote growth and avoid sickness. Due to the prolonged use of sub‐therapeutic quantities in feeds, macrolides may accumulate in the human body through the food chain, increasing the risk of the emergence of antibiotic resistance (Song et al. 2018; Wang 2009). The 16‐membered macrolide antibiotics (tylosin A and josamycin‐based) and 14‐ and 15‐membered (erythromycin‐based) drugs are mainly used in veterinary medicine. Tylosin is poorly biodegradable and can remain in animal food products such as milk, meat, eggs and honey (Arsic et al. 2018).

Residues from antibiotics classes of macrolides (erythromycin, azithromycin, tylosin, tilmicosin and spiramycin) can be identified in animal products by using simple and fast analysis methods (Jank et al. 2015).

### 3.6 Avermectins

The macrolide compounds known as avermectins, which include ivermectin, doramectin and eprinomectin, have also been widely utilized as anti‐parasitic medications to safeguard human, animal and agricultural health (Hua et al. 2021; Xie et al. 2022). These drugs are categorized as highly effective but toxic natural products isolated from *Streptomyces avermitilis* and are widely applied as anti‐helminthic and anti‐parasitic agents in agriculture and aquaculture (Qiao et al. 2022; Turnipseed and Andersen 2008).

### 3.7 Benzimidazoles

Benzimidazole anthelmintics (albendazole, fenbendazole, mebendazole, cambendazole and carbendazim) have been broadly used for the prevention and treatment of endo‐parasites in animals (Atta et al. 2022; Kim et al. 2021; Yoo et al. 2021). These groups of drugs exert their effects by inhibiting tubulin polymerization, progressively depleting energy reserves and inhibiting excretion of waste products and protective factors from parasite cells (Patel et al. 2018).

### 3.8 Non‐Steroidal Anti‐Inflammatory Drugs

Anti‐inflammatory medications that do not have steroid structures are referred to as NSAIDs. Due to their ability to conjugate with the isomers of cyclooxygenase, which converts arachidonic acid into prostaglandin, they are widely utilized in the livestock and poultry farming industries and have significant applications in the treatment of arthritic conditions, sports injuries and mammalian mastitis (Shishov, Nechaeva, and Bulatov 2019; Wang et al. 2019).

In contrast to their therapeutic effects, they may also have toxic and adverse consequences, such as gastrointestinal, hematopoietic or renal impacts. NSAIDs may also pose a risk to the public's health if these veterinary medications are found in animal products like milk and meat (van Pamel and Daeseleire 2015).

### 3.9 Aminoglycosides

Antibiotics known as aminoglycosides, such as streptomycin, gentamycin and neomycin are obtained from *Streptomyces* or *Micromonospora* species. This class of medications disrupts the protein synthesis of the majority of gram‐positive and gram‐negative bacteria to cause them harm (Turnipseed and Andersen 2008).

As aminoglycoside antibiotics are used in both medicine and agriculture most frequently, their mobility in the environment is increased, necessitating their detection in a variety of food matrices (Glinka, Wojnowski, and Wasik 2020). Abuse of these drugs in animal husbandry can result in antibiotic residues in milk, eggs and meat, and identification is important to protect the general public from the negative health effects of aminoglycosides (Yan et al. 2018). It is possible to recover aminoglycosides from a variety of animal‐derived products, such as beef, milk, pork, poultry muscle and eggs (Arsand et al. 2016; Lehotay and Lightfield 2018).

## 4 Causes of Veterinary Drug Residues

The occurance of drug residues in food products of animal origin may be due to miss use of antibiotics in animal's food, failure to observe the withdrawal period; the illegal use of antibiotics and the use of antibiotics as growth promoters; and failure to properly clean equipment used to mix or administer drugs (Arsène et al. 2022; Beyene and Tesega 2014). Due to poor control of drugs from the government authorities, information on the actual rational drug use pertaining to veterinary drug use is very limited. Food animals are slaughtered without screening, and no formal control mechanisms exist to protect the consumers against the consumption of meat and milk products containing harmful drug residues in developing countries. Some banned drugs in human medicine used in veterinary medicine/with no allowable extra‐label uses in any food‐producing animal species are chloramphenicol, clenbuterol, diethylstilbestrol, fluoroquinolone–class antibiotics, glycopeptides–all agents, including vancomycin, medicated feeds, nitroimidazoles, including dimetridazole, ipronidazole, metronidazole and others, nitrofurans–all agents, including furazolidone, and nitrofurazone (FARAD 2021; Ghimpețeanu et al. 2022).

## 5 Residues of Veterinary Drugs in Food Products of Animal Origin

### 5.1 Meat

Because of its abundant nutrient content and appealing sensory qualities, meat and meat products hold a particular place in our diet. But meat and animal products can easily get infected or spoiled, which makes them unsuitable (Biswas and Mandal 2020; Das et al. 2019). It is a significant issue when meat products include drug residues over the maximum allowable limits. These residues represent a health risk based on the maximum residue levels found in animal tissue and in relation to daily consumption of the public (Ramatla et al. 2017; Treiber and Beranek‐Knauer 2021).

Residues of ciprofloxacin (quinolones), amoxicillin (β‐lactams) and tetracyclines are found in the muscles of domestic chickens (Jammoul and El Darra 2019; Patel et al. 2018). Different levels of Ivermectin (a member of a group of naturally occurring macrocyclic lactones) residue are found in bovine liver (Jabber, Norian, and Jalilvand 2017). From another study in Turkey, 51.1% of quinolone antibiotic residue was detected from chicken meat and beef using enzyme‐linked immunosorbent assay (ELISA) (Er et al. 2013). Antibiotics were found in 28.6% of livestock and poultry meat samples collected from China using ultra‐performance liquid chromatography coupled to high‐resolution quadrupole time‐of‐flight mass spectrometry (QToF). Enrofloxacin and trimethoprim were the antibiotics that exceeded the maximum residue limits (MRLs) (Wang, Beier, and Shen 2017). Enrofloxacin and oxytetracycline were also detected in fishery products beyond their maximum residual limits in South Korea (Kang et al. 2018). Residues of ciprofloxacin (56%), streptomycin (34%), sulphanilamide (18%) and tetracycline (25.3%) were also detected from chicken, pork and beef muscles collected in South Africa (Ramatla et al. 2017). In a study conducted in Faisalabad, Pakistan, more than half, that is 22, (55%) of the beef samples exceeded the maximum (>100 ppb) residue limit for tetracycline (Qamar et al. 2023).

The use of veterinary drugs without respecting drug withdrawal period and lack of awareness about antimicrobial side effects contribute to the presence of antimicrobials like tetracyclines, β‐lactams and sulphonamides (76.4%) in beef from a study in North Western Ethiopia (Agmas and Adugna 2018).

### 5.2 Milk

Milk is consumed all around the world and is important both nutritionally and commercially. Despite being wholesome and nutritious, milk and milk products may nevertheless contain medication residues that could be dangerous to the public (Lourenco et al. 2020). Contaminated feed and water, improper veterinary drug use, careless milk withdrawal, poor milk collection and processing, and irresponsible milk withdrawal are all ways that residues might enter milk (Shaikh and Patil 2020). Antibiotic residues are primarily discovered in milk as a result of their careless use in treating infectious illnesses in animals. Additionally, some antibiotics are being indiscriminately utilized as feed additives, which is another source of antibiotic residues in milk and ultimately accountable for possible public health significance (Sachi et al. 2019). These medications persist in milk leftovers and have very negative effects on consumers, including cancer and allergic responses (Parmar et al. 2021). Sulphonamides are used most frequently to treat bovine mastitis, which results in medication residues in dairy products (Turnipseed and Andersen 2008). For instance, a study conducted by Qamar et al. (2023) in Faisalabad, Pakistan showed that more than half of the milk samples were contaminated with tetracycline residues and that the levels pose a serious health risk to consumers.

In Ethiopia, there are some reports showing the existence of veterinary drug residues in animal products. Oxytetracycline and penicillin G residue were found beyond the acceptable levels in milk collected from Central Ethiopia (Alredaisy 2011).

### 5.3 Eggs

The main antibiotic residue groups found in egg matrices include coccidiostats, anticoccidials, sulphonamides, nitrofurans, β‐lactams (amoxicillin) and macrolide group. This suggests that eating eggs poses a public health risk, particularly when antibiotic residue levels go above the MRLs. The usage of antibiotics may be essential given the rising global production rates of poultry eggs to satisfy the growing population's need for food and nutrition. Therefore, strong guidelines must be put in place to guarantee the safe or minimal use of antibiotics in poultry management (Owusu‐Doubreh, Appaw, and Abe‐Inge 2023). Veterinary drug residues of tetracycline (33.6%), quinolones (24.7%) and sulphonamides (4.8%) were recovered from poultry eggs collected from a study in China (Yang et al. 2020). Residues of sulfachloropyridazine, sulphamonomethoxine and fipronil sulphone were also detected by HPLC–QTOF‐MS (Hou et al. 2020).

### 5.4 Honey

Hence, honey bees can suffer from pests and diseases, so therapeutic drugs have been used to protect them from harmful effects of disease and pests. Tetracyclines, aminoglycosides, sulphonamides, macrolides and fluoroquinolones which are among the most used drugs in medication of honey bee diseases, usually result in high levels of residues in honey (Reybroeck 2018). Residues of acaricides used for sanitary treatments, coumaphos and two transformation products of amitraz (DMF and DMPF) were recovered from honey and other honey bee products by using liquid chromatography–tandem mass spectrometry (LC–MS/MS) method (Lozano et al. 2019).

#### 5.4.1 Residual Limits of Major Veterinary Drugs in Animal Products

The European Union is subject to the provisions of European Regulation 37/2010 on the maximum allowable limits of veterinary drug residues from food products of animal origin to protect the public from their adverse effects (European Union, 2010) Table 1.

**TABLE 1:** Maximum residual limits (MRLs) of veterinary drugs in products (European Union, 2010).

Active substance	Animal species	Target tissue	MRL (µg/kg)
Albendazole	All ruminants	Muscle	100
		Fat	100
		Liver	1000
		Kidney	500
		Milk	100
Amoxicillin	All food‐producing species	Muscle	50
		Fat	50
		Liver	50
		Kidney	50
		Milk	4
Ampicillin	All food‐producing species	Muscle	50
		Fat	50
		Liver	50
		Kidney	50
		Milk	4
Benzyl penicillin	All food‐producing species	Muscle	50
		Fat	50
		Liver	50
		Kidney	50
		Milk	4
Chlortetracycline	All food‐producing species	Muscle	100
		Liver	300
		Kidney	600
		Milk	100
		Eggs	200
Colistin	All food‐producing species	Muscle	150
		Fat	150
		Liver	150
		Kidney	200
		Milk	50
		Eggs	300
Cloxacillin	All food‐producing species	Muscle	300
		Fat	300
		Liver	300
		Kidney	300
		Milk	30
Diclofenac	Bovine	Muscle	5
		Fat	1
		Liver	5
		Kidney	10
		Milk	0.1
Doxycycline	Bovine	Muscle	100
		Liver	300
		Kidney	600
	Poultry and porcine	Muscle	100
		Skin and fat	300
		Liver	300
		Kidney	600
Enrofloxacin	Bovine, ovine, caprine	Muscle	100
		Fat	100
		Liver	300
		Kidney	200
		Milk	100
	Porcine, rabbit	Muscle	100
		Fat	100
		Liver	200
		Kidney	300
Enrofloxacin	Poultry	Muscle	100
		Skin and fat	100
		Liver	200
		Kidney	300
	All other food‐producing species	Muscle	100
		Fat	100
		Liver	200
		Kidney	200
Erythromycin	All food‐producing species	Muscle	200
		Fat	200
		Liver	200
		Kidney	200
		Milk	40
		Eggs	150
Fenbendazole	All ruminants, porcine, Equidae	Muscle	50
		Fat	50
		Liver	500
		Kidney	50
	All ruminants	Milk	10
Flunixin	Bovine	Muscle	20
		Fat	30
		Liver	300
		Kidney	100
	Porcine	Muscle	50
		Skin and fat	10
		Liver	200
		Kidney	30
Gentamicin	Bovine, porcine	Muscle	50
		Fat	50
		Liver	200
		Kidney	750
	Bovine	Milk	100
Ivermectin	All food‐producing mammals	Fat	100
		Liver	100
		Kidney	30
Kanamycin A	All food‐producing species except fin fish	Muscle	100
		Fat	100
		Liver	600
		Kidney	2500
		Milk	150
Oxytetracycline	All food‐producing species	Muscle	100
		Liver	300
		Kidney	600
		Milk	100
		Eggs	200
Streptomycin	All ruminants, porcine, rabbit	Muscle	500
		Fat	500
		Liver	500
		Kidney	1000
	All ruminants	Milk	200
Sulphonamides	All food‐producing species	Muscle	100
		Fat	100
		Liver	100
		Kidney	100
	Bovine, ovine, caprine	Milk	100
Tylosin A	All food‐producing species	Muscle	100
		Fat	100
		Liver	100
		Kidney	100
		Milk	50
		Eggs	200

## 6 Public Health Impact of Veterinary Drug Residues

Veterinary drug residues have essential public health consequences. Toxicity, allergic reaction, antimicrobial drug resistance, carcinogenicity, mutagenicity, teratogenicity and disruption of intestinal normal flora are among the major negative impacts of these drugs in the public (Sachi et al. 2019; Shaikh and Patil 2020; Yusuf and Firdisa 2022). The sustainability of diets is impacted by the overuse of veterinary antibiotics and synthetic growth boosters, which can also lead to an accumulation of residues in animal products and the environment (Gonzalez Ronquillo and Angeles Hernandez 2017). A study from China indicated that there was significant residue of veterinary antibiotics in food (Enrofloxacin, penicillin and erythromycin), water (oxytetracycline) and urine of pre‐school children (Norfloxacin and penicillin) (Li et al. 2017).

### 6.1 Antibiotic Resistance

An emerging global disease and a significant public health issue is the recent sharp rise in the proportion and absolute quantity of bacterial infections resistant to several antibacterial treatments (Roca et al. 2015). Due to the possibility of the development of antimicrobial resistance (AMR), the presence of antimicrobial residues in meat, milk and eggs poses a major risk to the public's health. It can have a negative effect on the food supply chain (Hassan et al. 2021; Ma et al. 2021; Serwecińska 2020). A global epidemic of AMR is a result of the careless and excessive use of therapeutically useful antibiotics in veterinary, medicinal and agricultural settings. Researchers and stakeholders are becoming increasingly concerned that the environment has been identified as an AMR reservoir and is crucial in the spread of genes linked with antibiotic resistance (Samreen et al. 2021).

Bacteria may adapt after receiving low doses of antibiotics, increasing their virulence and resistance. Positive selection, target change and spontaneous mutations are the main causes of this resistance acquisition, which leads to lower affinity and the development of resistance genes (Manyi‐Loh et al. 2018). The use of various antimicrobial substances in food animals, such as cephalosporins, macrolides and virginiamycin, may have an effect on human AMR. The micro‐organisms most frequently identified in agriculture and food are multidrug‐resistant *Escherichia coli*, resistant *Salmonella*, enterococci resistant to glycopeptides or streptogramins, and *Campylobacter* resistant to macrolides or fluoroquinolones (Ma et al. 2021; Owusu‐Doubreh, Appaw, and Abe‐Inge 2023; Samreen et al. 2021). For instance, studies in Ethiopia revealed that *E. coli* is the most common pathogen that showed resistance to broad‐spectrum antibiotics with the highest resistance in ampicillin (98%), amoxicillin‐clavulanate (90%) and co‐trimoxazole (trimethoprim/sulfamethoxazole) (77%) and *Staphylococcus aureus* isolates showed resistance to penicillin G (95%), cefoxitin (77.19%), tetracycline (63.41%), streptomycin (60.78%), gentamicin (59.37%), vancomycin (56.75%), clindamycin (54.35%) and bacitracin (53.65%) (EPHI 2019).

**FIGURE 1:**
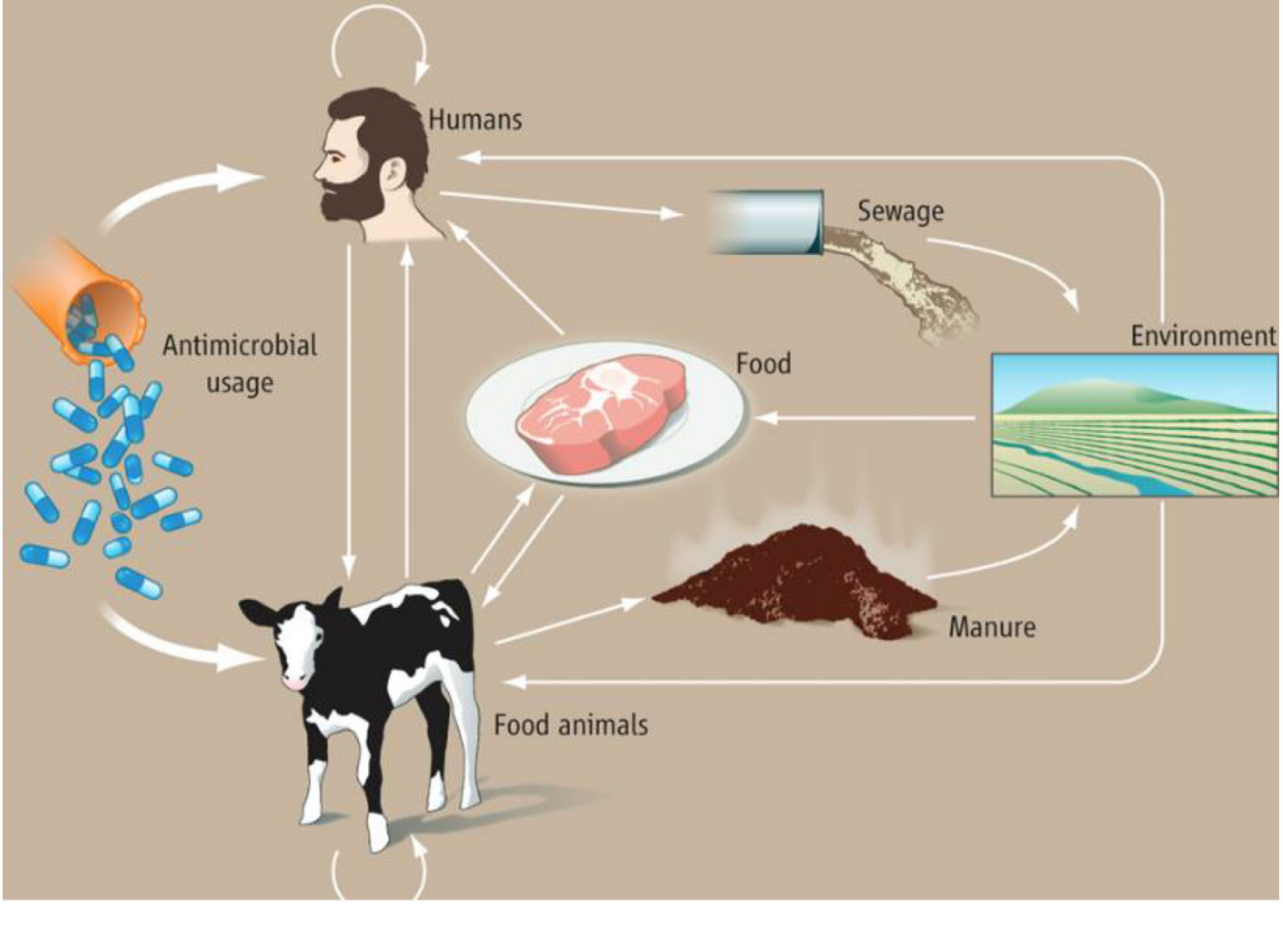
Routes of antimicrobial resistance transmission (animals, humans and environment) (McEwen and Collignon 2018).

Pathogenic bacteria strengthen their resistance to antibiotics by acquiring antimicrobial resistance genes (ARGs) through plasmid exchange at the gene level (Jian et al. 2021). These ARGs can travel through food animals, manure and wastewater and eventually find its way into the food supply chain (Figure 1) (Kirbis and Krizman 2015; McEwen and Collignon 2018). ARGs are present in bacteria primarily from farm animals. These genes are produced in animals and are widely employed in animal production, even though their use has decreased in certain nations. ARGs are mostly transmitted to humans through the consumption of foods of animal origin. When antibiotics are administered, bacteria in foods of animal origin may show resistance, which may be further selected for and amplified (De Alcântara Rodrigues et al. 2020; Kirbis and Krizman 2015).

Based on the class of antibiotics to which they confer resistance, resistance genes can be categorized into the following groups: β‐lactams (bla), tetracyclines (tet), sulphonamides (sul), macrolides (erm), aminoglycosides (aac), fluoroquinolone (fca), colistin (mcr), vancomycin (van) and multidrug (mdr) (Table 2) (Jian et al. 2021).

**TABLE 2:** Multidrug‐resistant pathogens and their resistant genes from animal sources.

Resistant pathogen	Resistant gene	Resistant to	References
*Salmonella*	*tetA*, *tetB*, *tetC, tetD*	Tetracycline, MDR	Adesiji, Deekshit, and Karunasagar (2014)
*Campylobacter*	*tet*O, *gyr*A	Fluoroquinolones, erythromycin	Kumar, Arnipalli, and Ziouzenkova (2020)
*Staphylococcus aureus*	*mec*A	Methicillin	Algammal et al. (2020); Bennani et al. (2020)
*Escherichia coli*	*aac*, *aad; tetA, tetB, tetC, and tetG; blaTEM, blaOXA‐20*	Aminoglycosides, tetracycline, β‐lactams	Kirbis and Krizman (2015); Xu et al. (2022); Qamar et al. (2023)
*Klebsiella pneumoniae*	*bla*CTX‐M‐15, *bla*OXA	β‐Lactams	Wareth and Neubauer (2021)
*Enterobacteriaceae*	*bla*IMP, *bla*VIM, *bla*NDM, *bla*OXA‐48	Carbapenems	Taggar et al. (2020)
*Pseudomonas*	*sul1, sul2, sul3*	Sulphonamides	Xu et al. (2022)

### 6.2 Toxicity (Direct and Indirect)

Humans may have gastrointestinal symptoms, allergic reactions and hepatotoxicity when macrolides are present in tissues from animals (Moga et al. 2021). Additionally, tetracyclines have been linked to gastrointestinal problems, teeth discolouration in young children, poor foetal development, pro‐inflammatory, cytotoxic and immuno‐pathological effects. High and frequent exposure to aminoglycoside residues in animal products can also cause ototoxicity and nephrotoxicity (Baynes et al. 2016). Drugs residues of thalidomide, albendazole, tetracyclines, aminoglycosides and hormones (diethylstilbestrol, misoprostol) beyond their level of maximum limits have been reported to cause toxic and teratogenicity effects to the foetus (Shaikh and Patil 2020).

### 6.3 Allergic Reactions

Antimicrobial drug residues provide particular safety issues for humans when it comes to allergies, which can range from rashes to potentially fatal anaphylactic reactions (Dowling 2013). Hypersensitivity reactions in humans may occur shortly after the consumption of food containing drug residues. Anaphylaxis, allergic skin reactions or delayed hypersensitivity responses are examples of allergic reactions to drugs (Ortelli, Spörri, and Edder 2018).

Tetracyclines have been reported to cause body reactions like allergy, skin rashes and phototoxic in humans (Bacanlı and Başaran 2019). β‐Lactams (penicillin and cephalosporin) are documented to cause dermatitis, cutaneous eruptions, anaphylaxis, haemolytic anaemia, vasculitis, acute interstitial nephritis and gastrointestinal symptoms in humans (Baynes et al. 2016; Paige, Tollefson, and Miller 1999).

### 6.4 Carcinogenic Effect

Residues from tetracycline, furazolidone, tamoxifen, phenobarbital and DDT act as a carcinogen and can produce various types of cancers (Shaikh and Patil 2020). From another report, carcinogenic effects have been also found with antibiotics such as sulphamethazine, oxytetracycline and furazolidone (Treiber and Beranek‐Knauer 2021).

## 7 Methods of Detection of Veterinary Drug Residues in Animal Products

A vital food safety concern is the quick identification and characterization of veterinary medication residues in foods with animal origin (Toldra and Reig 2006). Limiting the tolerances or maximum residual levels permitted for use in food animals is a crucial analytical challenge for protecting the public from potential health risks from these residues (Turnipseed and Andersen 2008). Currently, there are several approaches (including microbiological, immunological and physico‐chemical) methods for identifying and determining the presence of veterinary drug residues in food products of animal origin (e.g. thin‐layer chromatography, HPLC, LC–MS/MS) (Gaudin 2017).

### 7.1 Chromatography With Spectrophotometry

The identification of several veterinary drug residues in animal products is greatly facilitated by medium‐to‐high‐resolution mass spectrometers (M‐HRMS) (Gómez‐Pérez et al. 2015). Liquid chromatography–mass spectrometry (LC–MS) is the prevailing technique for assessing residues of veterinary drugs in animal products because LC offers a versatile and universal separation (Masiá et al. 2016). Recently, a more rapid with high selectivity and sensitivity method for detecting veterinary drugs based on LC–MS/MS and time‐of‐flight mass spectrometry (ToF/MS) is developed. LC‐IT‐ToF/MS can detect a large number of veterinary drug residues (more than 100) in a single run (Kang et al. 2017). To analyse the presence of various veterinary antibiotic residues as well as agricultural fungicides and insecticides in livestock and poultry products, researchers have developed an ultra‐high‐performance liquid chromatography–tandem mass spectrometry (UPLC–MS/MS) method based on selective accelerated solvent extraction and magnetic material purification (Wang et al. 2020).

### 7.2 Biosensors

Biosensors are innovative methods of screening antibiotics in food and many other analytical fields. Biosensor instruments include a bio‐receptor (bio‐recognition) element, which recognizes the target, and a transducer for converting the recognition event into a measurable signal (Gaudin 2017). Biosensors can detect enzymes, immunoglobulins, RNA/DNA aptamers, synthetic molecules and bacteria. Antigen/antibody (immunoglobulin)–based biosensors are the most frequently used biosensors for detecting veterinary drug residues in food products of animal origin (Majdinasab et al. 2017). Biosensors are emerging methods, applied in screening antibiotic residues in animal‐derived foods. They can deliver real‐time measurements, a high degree of automation and high throughput, less expensive and time‐saving for analysis (Chen et al. 2017).

Aptamer‐based biosensors and optical and electrochemical biosensors have been constructed and recently advanced for the detection of veterinary drugs in foods of animal origins (Xie et al. 2022). These technologies are expected to have more extensive research and application prospects in the fields of molecular diagnosis, food inspection, environmental monitoring and medical inspection (Huang 2020).

### 7.3 Immunological Methods

Immunological methods of veterinary drug residue analysis are based on the highly specific reaction of the substance with antibodies (Cui et al. 2018). ELISA and lateral flow immunoassay (LFIA) seem to be rapid and inexpensive methods of mass screening antibiotics in food products (Hendrickson et al. 2021; Wang, Beier, and Shen 2017). Immunoassays are convenient tools for screening a large number of samples due to their simplicity, accuracy and high‐throughput process (Acaroz et al. 2020).

## 8 One Health Concept to Combat Antimicrobial Residues in Animal Products

**FIGURE 2:**
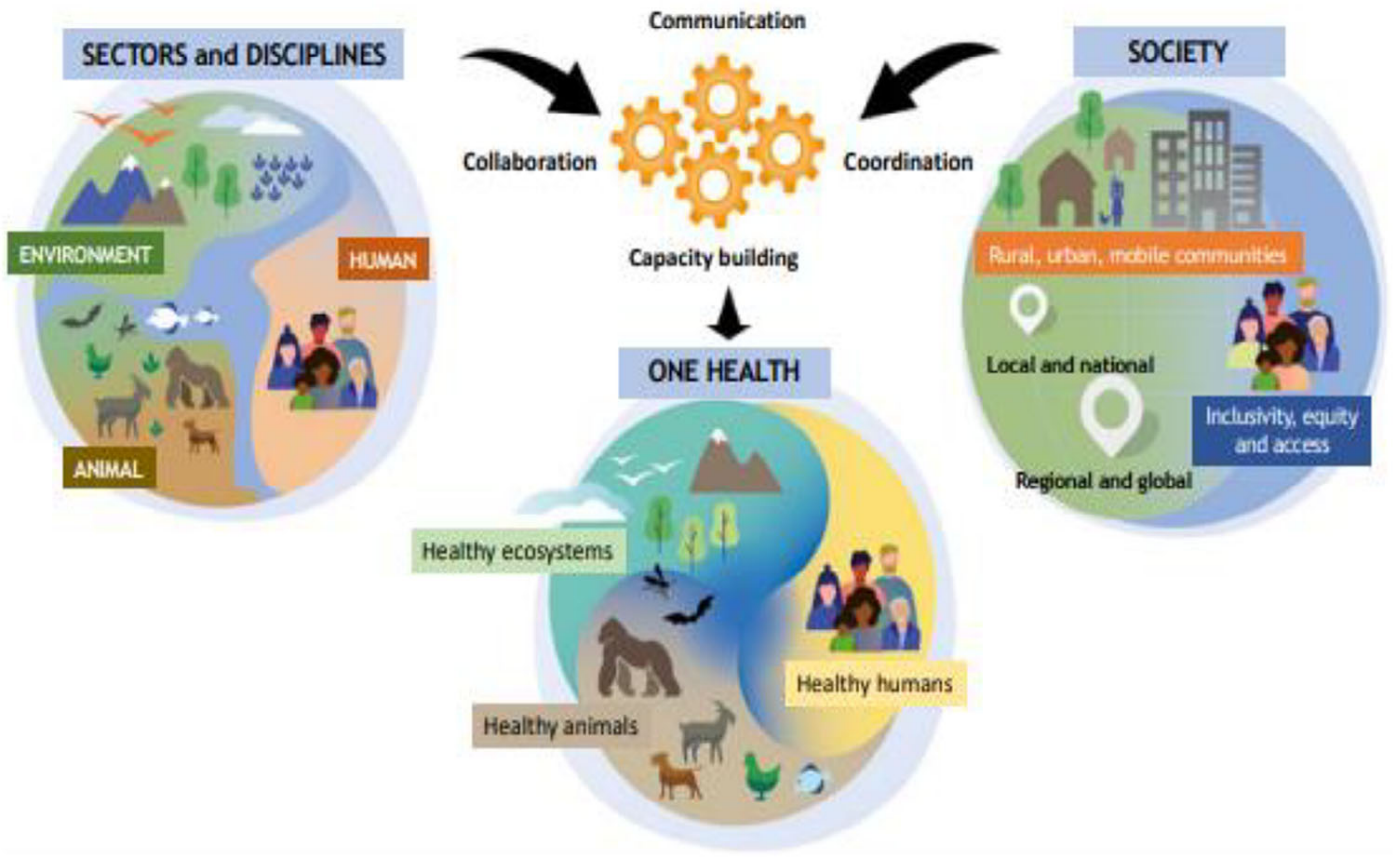
Definition of One Health. *Source*: Adisasmito et al. (2022).

One Health is the collaborative effort of multiple disciplines, working locally, nationally and globally, to attain optimal health for all: people, animals and our environment (Figure 2). Among the global health problems, antimicrobial residues leading to AMR are the ones that most clearly need the One Health approach (Ahmad, Joji, and Shahid 2023; Velazquez‐Meza et al. 2022). There is currently a global convergence around the need for greater intersectoral and multidisciplinary collaboration in addressing threats and reducing the risks of antimicrobial residues at the human–animal–ecosystem interface. World Health Organization, Food and Agriculture Organization of the United Nations, United Nations Environment Programme and World Organization for Animal Health collaborate and discuss on One Health priority research agenda of antimicrobial residues that lead AMR, sharing responsibilities and coordinating global activities to address this health risk at the human–animal–ecosystem interface (WHO, FAO, UNEP or WOAH 2023). Thus, One Health is more synergistic, integrated and holistic approach to systematically understanding the complex relationships among humans, animals and environments, which may provide effective countermeasures to solve animal‐derived food safety problems such as antimicrobial residues. Because of the connectivity of these sectors, antibiotic use and antibiotic residue persistence, thus the existence of antibiotic‐resistant bacteria in human–animal–environment habitats, are all linked to the One Health triangle. The pillars of support, including rigorous antimicrobial residue surveillance among different sectors individually and in combination, and at the national and international level, overcoming laboratory resource challenges, and core plan and action execution, should be strictly implemented to combat antimicrobial residues under One Health approach (Ahmad, Joji, and Shahid 2023; WHO, FAO, UNEP or WOAH 2023).

Thus, improve our understanding of antimicrobial residue on animal origin food, drivers and impact, strengthen the evidence base for interventions, advocate for the prioritization of antimicrobial residue mitigation and inform policy‐making in One Health approach is per amount important (WHO, FAO, UNEP or WOAH 2023).

The one that most exemplifies the One Health approach is AMR, a major worldwide issue that impacts people, the environment (water, air, soil or manure) and animals (Ma et al. 2021; Velazquez‐Meza et al. 2022). As AMR can spread quickly throughout the community, in healthcare settings, the food chain and the environment, managing many infectious diseases in humans and animals becomes more difficult (Tang, Millar, and Moore 2023). AMR dissemination should be limited through awareness creation and education about antibiotic use, policy promotion and advocacy on rational utilization of antimicrobial in food production system (Aslam et al. 2021). The main areas of concern at the moment are antimicrobial residues in food that could arise from the careless use of antibiotics in agriculture. Any step along the farm‐to‐table continuum might have AMRB contamination in food and food products (Samtiya et al. 2022).

## 9 Conclusion and Recommendations

### 9.1 Conclusion

The presence of veterinary drug residues in food products of animal origin is a serious public health and food safety concern. Numerous studies confirmed that there are abundant residues of veterinary drugs, mainly antibiotics in meat, milk, eggs and honey that have drawn attention to the unjustified use of antibiotics and the danger of consumer antibiotic resistance issues, which are propagated by foods containing antibiotic residues. There are also reports of toxicity, teratogenicity, hypersensitivity and carcinogenic reactions which are directly related to the consumption of veterinary medication remnants in animal products. Different conventional and advanced methods of detecting drug residues in food products revealed that antimicrobial can be detected in different concentrations and forms as residues in all food groups, including meat and meat products, milk and dairy products, eggs, honey and non‐animal‐origin goods. Despite the alarming signs of irrational utilization of veterinary drug and increasing their negative public health and environmental impact, advanced methods of determining residues and regulatory guidelines are not well implemented globally, especially in developing countries.

### 9.2 Recommendations

To minimize the level of veterinary drug residues in food products of animal origin and to reduce their public health consequence, the following recommendations are forwarded:

- Implementation of global integration in awareness creation, regular and efficient detection of residues.
- Strict implementation of food safety, environmental and public health rules and regulations for a better public health safety in the future.
- Scientific guidelines and precautions to minimize antibiotic residue will be strictly forwarded in the food of farm animals and discourage the advertisement of antibiotic as feed additives.
- Animal diseases and infections should primarily be prevented by ensuring biosecurity, following good production and good management practices.
- Knowledge and public awareness should be created on the public health risk of antibiotic residue.
- Further research should be conducted regarding antibiotic residue in food of animal origin in Ethiopia so that it may help in designing the mitigating approaches.

## Author Contributions

**Yeshambel Muluye Mesfin**: conceptualization, formal analysis, methodology, writing–original draft. **Birhan Agmas Mitiku**: conceptualization, methodology, resources, supervision, visualization, writing–original draft, writing–review and editing. **Habtamu Tamrat Admasu**: methodology, supervision, validation, visualization, writing–review and editing.

## Ethics Statement

The authors have nothing to report.

## Consent

The authors have nothing to report.

## Conflicts of Interest

The authors declare no conflicts of interest.

### Peer Review

The peer review history for this article is available at https://publons.com/publon/10.1002/vms3.70049.

## Data Availability

All data and material will be available upon requests to the corresponding author
